# Hydrocele of the Canal of Nuck in an Adult Woman: A Rare Cause of Inguinal Swelling

**DOI:** 10.7759/cureus.77682

**Published:** 2025-01-19

**Authors:** Sara F Alhajri, Lilian M Haji, Alhanouf H Hatim, Sajeda K Mohamed, Anas E Ahmed

**Affiliations:** 1 Alfaisal University, College of Medicine, Riyadh, SAU; 2 First Moscow State Medical University, College of Medicine, Moscow, RUS; 3 General Practice, King Fahad General Hospital, Jeddah, SAU; 4 Dentistry, Wenzhou Medical University, Whenzhou, CHN; 5 College of Medicine, Jazan University, Jazan, SAU

**Keywords:** adult presentation, canal of nuck, embryological processus vaginalis, hydrocele, inguinal swelling, pediatric patients

## Abstract

A hydrocele of the canal of Nuck is an uncommon condition resulting from incomplete closure of a developmental structure in females. While more frequent in children, it can also appear in adults, where its nonspecific presentation as an inguinal swelling may complicate diagnosis. We report the case of a 36-year-old woman who presented with a painless, gradually enlarging swelling in the right inguinal region. Physical examination revealed a soft, fluctuant, non-reducible swelling confined to the inguinal canal. Imaging studies, including ultrasound, computed tomography, and magnetic resonance imaging, demonstrated a well-circumscribed, fluid-filled lesion consistent with a hydrocele of the canal of Nuck. The patient underwent a successful surgical excision, and a histopathological examination confirmed the diagnosis of a benign cystic lesion. She had an uneventful recovery with no recurrence at the six-month follow-up. This case emphasizes the need for heightened clinical awareness of hydrocele of the canal of Nuck, particularly in adult women presenting with inguinal swelling. Timely diagnosis and management can ensure excellent patient outcomes while avoiding unnecessary interventions.

## Introduction

The canal of Nuck is an embryological structure in females that is analogous to the processus vaginalis in males. During fetal development, it descends with the round ligament into the inguinal canal and normally obliterates shortly after birth. Failure of this obliteration can lead to the persistence of a peritoneal sac, predisposing to the formation of conditions such as hydrocele, hernias, or other inguinal pathologies [1,2]. A hydrocele of the canal of Nuck is a rare condition characterized by the accumulation of serous fluid within this remnant. Though predominantly asymptomatic, it can present as an inguinal swelling, often leading to diagnostic confusion with hernias, lymphadenopathy, or cystic neoplasms [2,3].

The condition is more frequently recognized in pediatric patients, but it is underdiagnosed in adults due to its rarity and nonspecific presentation [2,3]. Imaging modalities such as ultrasound, computed tomography, and magnetic resonance imaging play pivotal roles in differentiating the hydrocele of the canal of Nuck from other inguinal masses. Surgical excision remains the definitive treatment, offering both therapeutic resolution and confirmation of diagnosis via histopathology [3,4]. This case underscores the importance of considering this entity in adult females presenting with inguinal swelling to avoid misdiagnosis and ensure appropriate management.

## Case presentation

A 36-year-old woman presented with a complaint of a painless swelling in the right inguinal region. She reported first noticing the swelling approximately two months prior, with a gradual increase in size. The swelling was non-tender and did not fluctuate in size with physical activity or changes in position. There was no associated erythema, warmth, or overlying skin changes. The patient denied any systemic symptoms, including fever, weight loss, or night sweats. She had no significant medical or surgical history, no history of trauma to the area, and no known allergies. Her menstrual cycles were regular, and she had no history of pregnancies or gynecological issues. There was no family history of similar conditions or hernias.

On physical examination, a non-tender, soft, and fluctuant swelling was noted in the right inguinal region. The swelling measured approximately 5 × 3 cm and was confined to the inguinal canal. There was no associated erythema, warmth, or signs of inflammation. The swelling did not reduce upon lying down or with gentle manual pressure, and it did not exhibit transillumination. There was no cough impulse. Examination of the left inguinal region and contralateral structures was unremarkable. Systemic examination revealed no abnormalities.

Routine laboratory investigations, including complete blood count, basic metabolic panel, and inflammatory markers (C-reactive protein and erythrocyte sedimentation rate), were within normal limits. Tumor markers, including CA-125, alpha-fetoprotein, and beta-human chorionic gonadotropin, were also normal (Table 1).

**Table 1:** Laboratory investigations and results

Test	Result	Reference range
White blood cell count	6.2 x 10³/μL	4.0–11.0 x 10³/μL
Hemoglobin	13.8 g/dL	12.0–15.5 g/dL
Platelets	250 x 10³/μL	150–450 x 10³/μL
Sodium	140 mmol/L	135–145 mmol/L
Potassium	4.2 mmol/L	3.5–5.0 mmol/L
Creatinine	0.8 mg/dL	0.6–1.1 mg/dL
Blood urea nitrogen	14 mg/dL	7–20 mg/dL
C-reactive protein	< 1.0 mg/L	< 3.0 mg/L
Erythrocyte sedimentation rate	10 mm/hr	0–20 mm/hr (female)
CA-125	15 U/mL	0–35 U/mL
Alpha-fetoprotein	2.1 ng/mL	0–8 ng/mL
Beta-human chorionic gonadotropin	< 1.0 IU/L	< 5.0 IU/L

**Figure 1:**
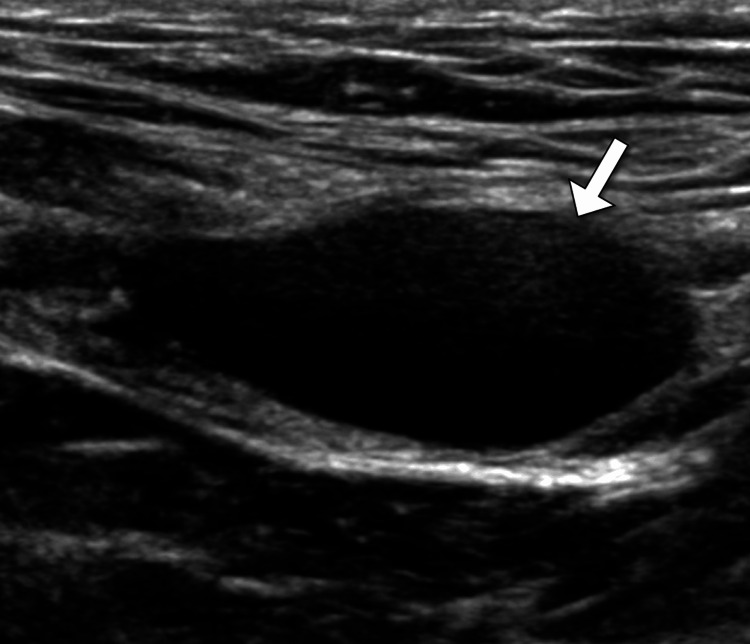
Ultrasound finding of the swelling Ultrasound examination revealed a well-defined anechoic structure without internal components, consistent with a cyst.

**Figure 2:**
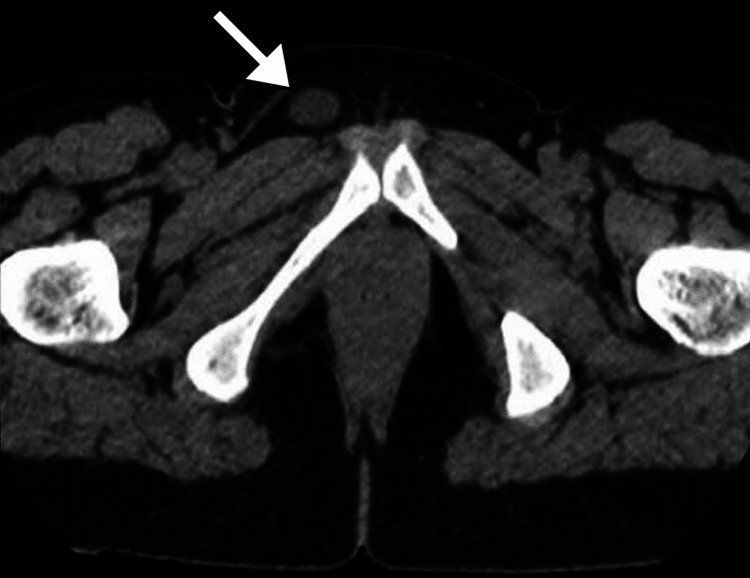
CT image of the pelvis Selected axial images from the CT pelvis scan demonstrated a well-defined, fluid-filled lesion (arrows) in the right inguinal canal, with no evidence of calcification or soft tissue components. CT: computed tomography

**Figure 3:**
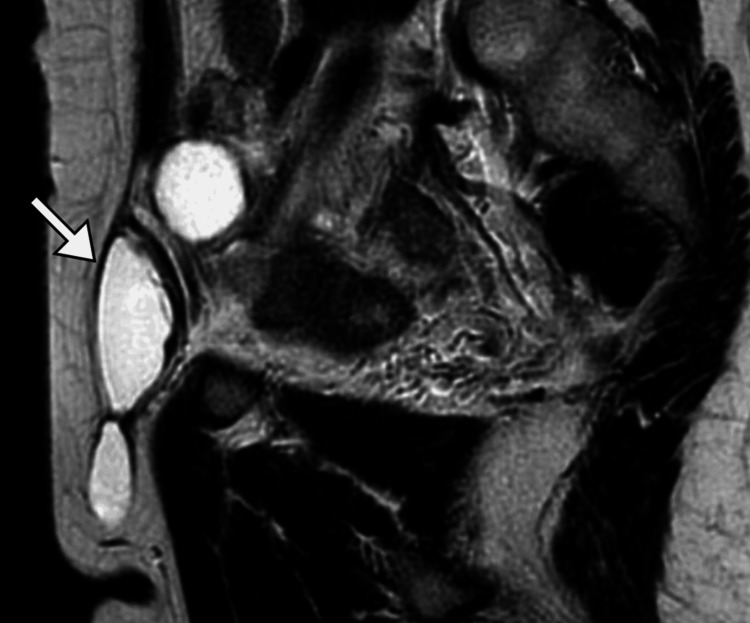
MRI image of the pelvis Selected sagittal MRI image of the pelvis showed a lesion with fluid signal intensity (arrow) in the right inguinal canal. MRI: magnetic resonance imaging

Ultrasound examination revealed a well-defined cystic structure without internal components (Figure 1). A computed tomography (CT) scan of the abdomen and pelvis with contrast confirmed a well-circumscribed, fluid-filled lesion in the right inguinal canal, consistent with a hydrocele. The surrounding structures, including the ovaries and uterus, appeared normal (Figure 2). Magnetic resonance imaging (MRI) of the pelvis with gadolinium contrast provided further characterization, showing a hyperintense, well-defined cystic structure on T2-weighted images. The lesion had a thin wall without enhancement, consistent with a hydrocele of the canal of Nuck. No evidence of adjacent tissue invasion or malignancy was noted (Figure 3).

The patient was counseled regarding the diagnosis of hydrocele of the canal of Nuck, a rare congenital condition resulting from the failure of the processus vaginalis to close. Given the absence of symptoms such as pain or complications, conservative management was initially considered. However, the patient opted for surgical intervention due to cosmetic concerns and the potential risk of complications.

The patient underwent elective surgical excision of the hydrocele via an inguinal approach under general anesthesia. Intraoperative findings confirmed a cystic structure confined to the inguinal canal without communication with the peritoneal cavity. The hydrocele was carefully dissected and excised.

The patient had an uneventful postoperative course and was discharged on the first postoperative day. At the two-week follow-up, the surgical site was well-healed, and the patient reported complete resolution of the swelling with no recurrence of symptoms. A six-month follow-up demonstrated no recurrence on clinical examination. The patient expressed satisfaction with the outcome.

## Discussion

A hydrocele of the canal of Nuck is a rare congenital anomaly resulting from the incomplete obliteration of the processus vaginalis in females. This condition is analogous to a hydrocele in males, but its rarity and nonspecific presentation often lead to diagnostic delays or misdiagnoses [3-5]. Although benign in nature, the hydrocele of the canal of Nuck warrants careful evaluation due to the potential for confusion with other inguinal pathologies, including hernias, lymphadenopathy, or neoplastic processes [2]. This discussion explores the embryological basis, clinical presentation, diagnostic approach, treatment, and significance of this condition.

During embryogenesis, the canal of Nuck forms as a tubular extension of the peritoneum that descends with the round ligament into the inguinal canal. Normally, this structure obliterates postnatally, leaving only a fibrous remnant. Persistent patency of the canal creates a potential space for fluid accumulation, resulting in a hydrocele [3,5]. The hydrocele may be classified as communicating or non-communicating based on its continuity with the peritoneal cavity. Non-communicating hydroceles, as observed in this case, result from isolated fluid accumulation without communication with the abdominal cavity. The rarity of hydrocele of the canal of Nuck in adult women is likely due to the higher likelihood of spontaneous closure of the canal during childhood [3,4].

Patients with hydrocele of the canal of Nuck typically present with a painless, unilateral swelling in the inguinal region. The swelling is usually soft, fluctuant, and non-tender, as seen in our patient. Unlike inguinal hernias, hydroceles do not exhibit a cough impulse or reducibility. The absence of overlying skin changes or systemic symptoms further distinguishes this condition from lymphadenopathy or infectious processes [1-4]. Differentiating it from other cystic or solid lesions, such as lipomas, abscesses, or tumors, is critical to avoid unnecessary interventions or delays in treatment.

Accurate diagnosis requires a combination of clinical evaluation and imaging studies. Ultrasound is the first-line modality due to its accessibility, cost-effectiveness, and ability to differentiate cystic from solid lesions. The anechoic appearance of the lesion on ultrasound, coupled with the absence of vascularity on Doppler imaging, supports the diagnosis of a benign cystic structure. Cross-sectional imaging offers superior anatomic detail and aids in ruling out malignancy or complex pathology.

The definitive treatment of hydrocele of the canal of Nuck is surgical excision, which addresses both diagnostic uncertainty and the potential for complications, such as infection, rupture, or cosmetic concerns [5,6]. An inguinal approach allows for the complete removal of the hydrocele and the preservation of surrounding structures. Histopathological examination of the excised lesion is essential to confirm the diagnosis and exclude rare cases of malignancy [2,4]. In this patient, surgical excision provided resolution of symptoms, and histopathology confirmed a benign cystic lesion.

The prognosis following surgical treatment is excellent, with minimal risk of recurrence when complete excision is achieved. Postoperative complications are rare and generally limited to mild, self-limiting issues such as seroma or wound dehiscence. Long-term follow-up is typically unnecessary unless new symptoms arise [7-9].

Awareness of this rare condition is essential for clinicians evaluating inguinal masses in women. Misdiagnosis or delayed diagnosis can lead to unnecessary interventions, patient anxiety, or progression to complications. This case underscores the importance of integrating clinical findings with imaging results to establish a prompt and accurate diagnosis. Additionally, it highlights the role of interdisciplinary collaboration among primary care providers, radiologists, and surgeons in managing rare and unusual conditions effectively.

## Conclusions

In conclusion, a hydrocele of the canal of Nuck, though rare, should be considered in the differential diagnosis of inguinal swelling in females. Early recognition and accurate diagnosis, supported by clinical evaluation and imaging, are crucial to avoid unnecessary interventions and ensure optimal patient outcomes. Surgical excision remains the definitive treatment, providing both diagnostic clarity and symptom resolution. With timely management, the prognosis is excellent, and recurrence is rare. This case emphasizes the importance of clinical awareness and interdisciplinary collaboration to effectively manage this uncommon condition and improve patient care.
